# Telemedicine for Stroke: Quantifying the Long-Term National Costs and Health Benefits

**DOI:** 10.3389/fneur.2021.804355

**Published:** 2022-06-20

**Authors:** Lan Gao, Elise Tan, Joosup Kim, Christopher F. Bladin, Helen M. Dewey, Kathleen L. Bagot, Dominique A. Cadilhac, Marj Moodie

**Affiliations:** ^1^Deakin Health Economics, Institute for Health Transformation, Deakin University, Geelong, VIC, Australia; ^2^Stroke and Ageing Research, School of Clinical Sciences at Monash Health, Monash University, Melbourne, VIC, Australia; ^3^Stroke Division, Florey Institute of Neuroscience and Mental Health, Parkville, VIC, Australia; ^4^Ambulance Victoria, Doncaster, VIC, Australia; ^5^Eastern Health and Eastern Health Clinical School, Monash University, Melbourne, VIC, Australia

**Keywords:** stroke telemedicine, cost-effectiveness analysis, thrombolysis (tPA), long-term, ischemic stroke

## Abstract

**Objective:**

Few countries have established national programs to maximize access and reduce operational overheads. We aimed to use patient-level data up to 12 months to model the potential long-term costs and health benefits attributable to implementing such a program for Australia.

**Methods:**

A Markov model was created for Australia with an inception population of 10,000 people with stroke presenting to non–urban or suburban hospitals without stroke medical specialists that could receive stroke telemedicine under a national program. Seven Markov states represented the seven modified Rankin Scale (mRS) scores (0 no disability to 6 dead) plus an absorbing state for all other causes of death. The literature informed inputs for the model; for the telemedicine program (including program costs and effectiveness) and patients, these were extrapolated from the Victorian Stroke Telemedicine (VST) program with the initial status of patients being their health state at day 365 as determined by their mRS score. Costs (2018 Australian dollars, healthcare, non–medical, and nursing home) and benefits were reported for both the societal and healthcare perspectives for up to a 25 years (lifetime) time horizon.

**Results:**

We assumed 4,997 to 12,578 ischemic strokes would arrive within 4.5 h of symptom onset at regional hospitals in 2018. The average per person lifetime costs were $126,461 and $127,987 from a societal perspective or $76,680 and $75,901 from a healthcare system perspective and benefits were 4.43 quality-adjusted life years (QALYs) and 3.98 QALYs gained, respectively, for the stroke telemedicine program and practice without such program. The stroke telemedicine program was associated with a cost saving of $1,526 (from the societal perspective) or an additional $779 (from the healthcare system perspective) and an additional 0.45 QALY gained per patient over the lifetime. The incremental costs of the stroke telemedicine program ($2,959) and management poststroke ($813) were offset by cost savings from rehospitalization (–$552), nursing home care (–$2178), and non–medical resource use (–$128).

**Conclusion:**

The findings from this long-term model provide evidence to support ongoing funding for stroke telemedicine services in Australia. Our estimates are conservative since other benefits of the service outside the use of intravenous thrombolysis were not included.

## Introduction

Despite dramatic improvements in the treatment of acute stroke over the past 5 years, stroke remains the leading cause of disability and death of an adult in Australia (1) and worldwide, contributing to 5.5 million deaths (2). With timely access to evidence-based treatment, including intravenous thrombolysis and endovascular thrombectomy, the morbidity and mortality poststroke can be significantly reduced. However, the dispersed distribution of the Australian population, similar to other large countries, disadvantages people who live outside metropolitan areas in terms of access to diagnosis and treatment (3). For example, as a standard treatment for acute ischemic stroke, intravenous thrombolysis is generally considered and applied for patients presenting within 4.5 h of symptom onset (4). However, meeting such strict onset to needle times remains a challenge for patients residing in regional areas of Australia due to longer transport times to a hospital with thrombolysis capability. Moreover, the incidence of stroke is greater (117 vs. 100 per 100,000 population) among people living in regional areas than those in metropolitan Australia (2), which creates a strong need for improving access to specialist stroke care in regional areas.

One method for increasing access to stroke specialists in regional areas is *via* the use of telemedicine programs (5). Telestroke (or stroke telemedicine) programs aim to improve stroke outcomes for people living in regional areas by providing access to neurologists *via* real-time consultations to expedite diagnosis and treatment decisions in partnership with local clinical teams (6). A recent systematic review of eight economic evaluations of stroke telemedicine programs conducted in different countries suggested that such programs are generally cost-effective with favorable health gains (5). The limitation of the past study is that it has usually relied on simulation modeling using indirect sources of data. In our recent study, we have shown that the Victorian Stroke Telemedicine (VST) program in Australia is cost-effective [nominally lower costs telemedicine $98,777 vs. control $110,861 and greater benefits in terms of quality-adjusted life year (QALY) gains 0.53 vs. 0.38, per patient] within the first 12 months of stroke using patient-level data collected from 2014 to 2017 (7). The long-term benefits of the stroke telemedicine programs have been explored for the US and Europe (8) and for Australia, it remains unknown. Given the objective of scaling-up the stroke telemedicine programs nationally, quantifying the potential long-term costs and health benefits is needed to inform funding decisions and ensure rational resource allocation.

This research study aims to provide an economic evaluation of the potential long-term cost and benefits of an established stroke telemedicine program using the data-rich VST program as the case study.

## Methods

### Population

The modeled population was defined according to the original VST study (7). This was a primarily historical controlled, real-world cohort study, which compared a 12-months period prior to implementation to the first 12 months of implementation of the stroke telemedicine program in 16 participating hospitals in Victoria. We prospectively collected 3- and 12-month resource utilization and health outcome data from a sample of the control and intervention groups to inform the cost-effectiveness model. A detailed description of the study population has been reported elsewhere (6, 9). In brief, patients with a mean age of 74 years, 55% being male, were defined accordingly for the long-term simulation starting from the first day of the index stroke symptoms.

### Long-Term Simulation Model

**Figure 1:**
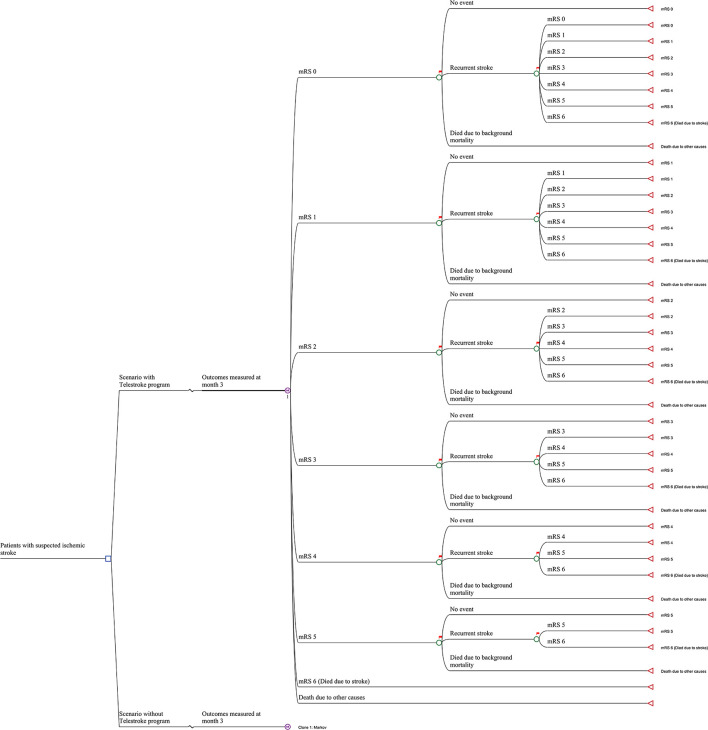
Model structure.

A Markov model was constructed to evaluate the long-term cost-effectiveness of the stroke telemedicine program vs. the care provided to patients with stroke in the absence of such a program (10, 11). Seven Markov states represented the seven modified Rankin Scale (mRS) scores (0 to 6) plus an absorbing state for all other causes of death. The initial status of patients in the model was their health state at day 365, as determined by their mRS score, informed by the VST study (7). From day 366 onward over the rest of their lifetime, patients who survived the first 365 days after their index stroke could experience a recurrent stroke or background mortality. Those who experienced recurrent stroke assumed that they could only transit to a health state that was equal to, or worse than, their current one and were unable to return to a better health state (e.g., moving from mRS 2 to mRS 1 was excluded). A similar model structure has been adopted for other economic evaluations of stroke treatments and the health states considered capturing the long-term costs and health outcomes associated with stroke (12). The long-term modeling was conducted using TreeAge software (Williamstown, Massachusetts, USA). The model structure is shown in Figure 1. The reference sources for long-term events are given in Table 1 and additional details are provided below.

**Table 1:** Inputs for the long-term cost-effectiveness analysis.

**Variable**	**Base case value**	**Reference**
**Transition probabilities**		
Probability of recurrent stroke	Year 1: 0.0649; Year 2+: 0.0201	Mohan et al. (13)
		Pennlert et al. (14)
Probability of death following a recurrent stroke^∧^	0.1783	Fagan et al. (43) Chen et al. (44)
Relative risk of having recurrent stroke^*^	1.48	Park et al. (16)
Probability of utilizing non-medical care		Gao et al. (19)
mRS 1	0.9138	
mRS 2	0.8431	
mRS 3	0.9070	
mRS 4	0.8963	
mRS 5	0.9232	
**HR of background mortality**		Hong et al. (45)
mRS 0	1.53	
mRS 1	1.52	
mRS 2	2.17	
mRS 3	3.18	
mRS 4	4.55	
mRS 5	6.55	
**Costs**		
Cost of rehospitalisation		IHPA 2018-19
mRS 0	$10,886	
mRS 1	$10,086	
mRS 2	$16,662	
mRS 3	$16,662	
mRS 4	$22,086	
mRS 5	$22,086	
mRS 6	$10,886	
Cost of management		
mRS 0	Year 1: $10,499; Year 2: $1,431	Arora et al. (10); Gloede et al. (17); Baeten et al. (18)
mRS 1	Year 1: $13,230; Year 2: $1,814	
mRS 2	Year 1: $15,943; Year 2: $1,814	
mRS 3	Year 1: $17,540; Year 2: $1,814	
mRS 4	Year 1: $20,772; Year 2: $1,814	
mRS 5	Year 1: $24,169; Year 2: $1,814	
Cost of non-medical care		Gao et al. (19)
mRS 1	$1,318	
mRS 2	$2,231	
mRS 3	$5,430	
mRS 4	$6,552	
mRS 5	$24,420	
Cost of nursing home care		Government website (46)
mRS 4	$40,689	
mRS 5	$40,689	
**Utility weights**		
mRS 0	0.836	Kim et al. (7)
mRS 1	0.777	
mRS 2	0.694	
mRS 3	0.437	
mRS 4	0.242	
mRS 5	0.064	

*mRS, modified Rankin Scale; $, Australian dollars; IHPA, Independent Hospital Pricing Authority Australia*.

∧ *0.11, for sensitivity analysis*.

* *The average RR was used*.

### Model Inputs

#### Transition Probabilities

The mRS scores at day 365 post the index stroke by the telestroke group or control (no telestroke access) were sourced from the original study (7). The only difference in transition probabilities between the two groups was the proportion of patients commencing the long-term simulation in each of the six health states (i.e., the mRS scores of 0–5; patients who died in the first 365 days were excluded from the long-term modeling, but their initial costs up to 12-months were included) as defined by the patient-level data. The annual probability of recurrent stroke (13, 14) and background mortality (15) was identical for the two treatment arms but was adjusted by the occurrence of recurrent events (i.e., experiencing a recurrent stroke leads to the increased risk of a subsequent one) (16).

The transition probabilities for both groups are shown in Table 1.

#### Costs

All the costs related to medical care, nonmedical care (i.e., nonmedical including accommodation changes, community services, home modifications, special equipment, informal care, etc.), and formal care (i.e., institutional care) were considered in the model (Table 1 and Supplementary Table 1) from a societal perspective starting from day 1 of the index stroke, while the medical care costs were included in the healthcare system perspective (10, 17–19). Productivity losses (i.e., paid work) were not considered, given the average age of the modeled population was 74 years and the retirement age set at 66 years in Australia (20). Costs associated with each health state, including the first 12 months (informed by the original study), recurrent hospitalizations, outpatient care (consultations, pharmaceuticals, and examinations), nursing home care (for patients with the mRS score 4 to 5), and non–medical care, were sourced from published literature (10). All the costs were valued in 2018 Australian dollars.

#### Utility Weights

Utility weights are preference weights representing the strength of desirability toward different health states (i.e., more preferred health states will have greater weight such as perfect health). They are measured on a cardinal scale of 0–1, where 0 indicates a health state equivalent or equal to death and 1 indicates perfect health (negative values represent a health state worse than death) (21). In this study, utility weights associated with being in the poststroke health states defined by the mRS score were directly informed by the VST participants measured using EQ-5D−3L at 12 months and were assumed to remain unchanged over the long term (22). The utility weights applied are shown in Table 1.

### Cost-Effectiveness Analysis

In the base case, 10,000 patients with stroke from regional/rural areas of Australia arriving within 4.5 h of symptom onset were simulated. A societal perspective was taken to provide a broader range of costs and health benefits (QALYs) over a 25 years time horizon. In addition, a healthcare system perspective was adopted to estimate the direct healthcare costs only and benefits for the same time horizon. Utility weights were used from the published literature to estimate the QALYs gained (21). In addition, life years lived were estimated to measure the survival gains. The primary outcome for the cost-effectiveness analysis was the incremental cost-effectiveness ratio (ICER) per QALY gained, which is calculated as the ratio between incremental cost and incremental QALYs gained (intervention vs. control). Costs and benefits were discounted at a rate of 3% per annum (23). The often-quoted willingness to pay (WTP) per QALY threshold of AU$50,000 was adopted to assess the potential cost-effectiveness of the stroke telemedicine program against a scenario with the no stroke telemedicine program (24).

### Sensitivity Analyses

One-way deterministic sensitivity analyses were conducted by varying one model parameter at a time within a plausible range to examine the robustness of base case results (Table 1). The results of a range of individual deterministic sensitivity analyses are combined and presented in the form of a tornado diagram (i.e., by showing the range of change in ICER). The probabilistic sensitivity analyses (PSAs), which determined the distribution of key uncertain parameters, were run to further explore the results. Additionally, the mRS outcomes at 1 year for the participants of the stroke telemedicine program were tested with a Dirichlet distribution. The Australian Telestroke Network study informed the distribution of the cost related to the stroke telemedicine program delivery (Supplementary Table 2). A key assumption made in the PSA was that the distributions for each parameter were not correlated (i.e., the variation in one parameter was not associated with the change in another parameter). An incremental cost-effectiveness plane was generated to illustrate the results of the PSA.

In order to identify the cutoff for the population size enabling the cost-effective implementation of the stroke telemedicine program, a threshold analysis was undertaken. The intervention cost was defined as the total intervention implementation cost ($1,762,892 from the primary study where all the costs for equipment in the first year operational model are incurred) divided by the population in a region.

### Impact of the National Implementation

The VST program directly informed the cost of stroke telemedicine delivery. The per capita intervention cost was conservatively assumed to remain the same during the national implementation even though the scaling-up is likely to result in lower per capita cost from improved efficiency. In order to quantify the long-term implications of implementing the national stroke telemedicine program, the annual numbers of patients were estimated for Australia in 2018 [the total number of strokes was 38,055 in 2018 (1)]. Based on the latest national acute stroke audit in Australia, of 4,176 patient case notes audited, 83% of strokes were ischemic (25). Among the ischemic strokes, approximately 60% were among people residing in the metropolitan area, while the balance of 40% comprised people from either inner regional or outer regional areas (26). We examined scenarios where 20%, 50%, and 100% of patients from inner regional (people from inner regional areas may be transferrable to a metropolitan hospital directly) would be eligible for the stroke telemedicine program, in addition to 100% of people from outer regional areas.

## Results

### Cost-Effectiveness Analysis

Overall, implementing the stroke telemedicine program for patients with ischemic stroke from regional Australia in the long term was a dominant strategy (more health gains and cost saving) from the societal perspective or highly cost-effective (ICER of $1,736 per QALY gained) from the healthcare system perspective.

The base case cost-effectiveness analysis showed that implementing stroke telemedicine in the long term was associated with lower costs and greater benefits. In particular, the average per patient lifetime costs for the stroke telemedicine program vs. the no stroke telemedicine program were $126,461 and $127,987 from a societal perspective or $76,680 and $75,901 from the healthcare system perspective. Corresponding benefits were 4.43 QALYs and 3.98 QALYs gained or 7.68 life years and 7.15 life years for the stroke telemedicine program and practice without such program, with a cost saving of $1,526 (from the societal perspective) or an additional $779 (from the healthcare system perspective) in cost and an addition 0.45 QALY gained per patient. The incremental cost of the stroke telemedicine program ($2,959) and management poststroke ($813) was partially offset by the cost savings arising from reduced rehospitalization (–$552), nursing home care (–$2178), and non–medical resource use (–$128) costs (Table 2).

**Table 2:** The results of base cost-effectiveness analysis.

	**Intervention**	**Control**	**Difference**
Total QALYs	4.428	3.979	0.449
Total LYs	7.687	7.145	0.542
**Societal perspective**			
Total costs	$152,209	$152,607	–$397
		**ICER (per QALY)**	**Dominant**
**Healthcare system perspective**			
	$102,429	$100,520	$1,908
		**ICER (per QALY)**	**$4,252**
Number of patients received nursing home care^*^	2861	2962	101
**Cost components**			
First 12-month cost	$78,859	$76,954	$1,904
Management cost	$11,267	$10,454	$813
Rehospitalisation cost	$12,303	$13,113	-$809
Nursing home cost	$46,590	$48,769	-$2,178
Non-medical cost	$3,190	$3,318	-$128

*QALY, quality-adjusted life year; LY, life year; ICER, incremental cost-effectiveness ratio*.

* *Per 10,000 patients over the lifetime. Dominant means less costs and more benefits*.

In addition, the stroke-related disability prevented due to the stroke telemedicine program was associated with a reduced probability of utilizing nursing home care poststroke, with a corresponding reduction of 101 nursing home residents per 10,000 patients.

### Sensitivity Analysis

**Figure 2:**
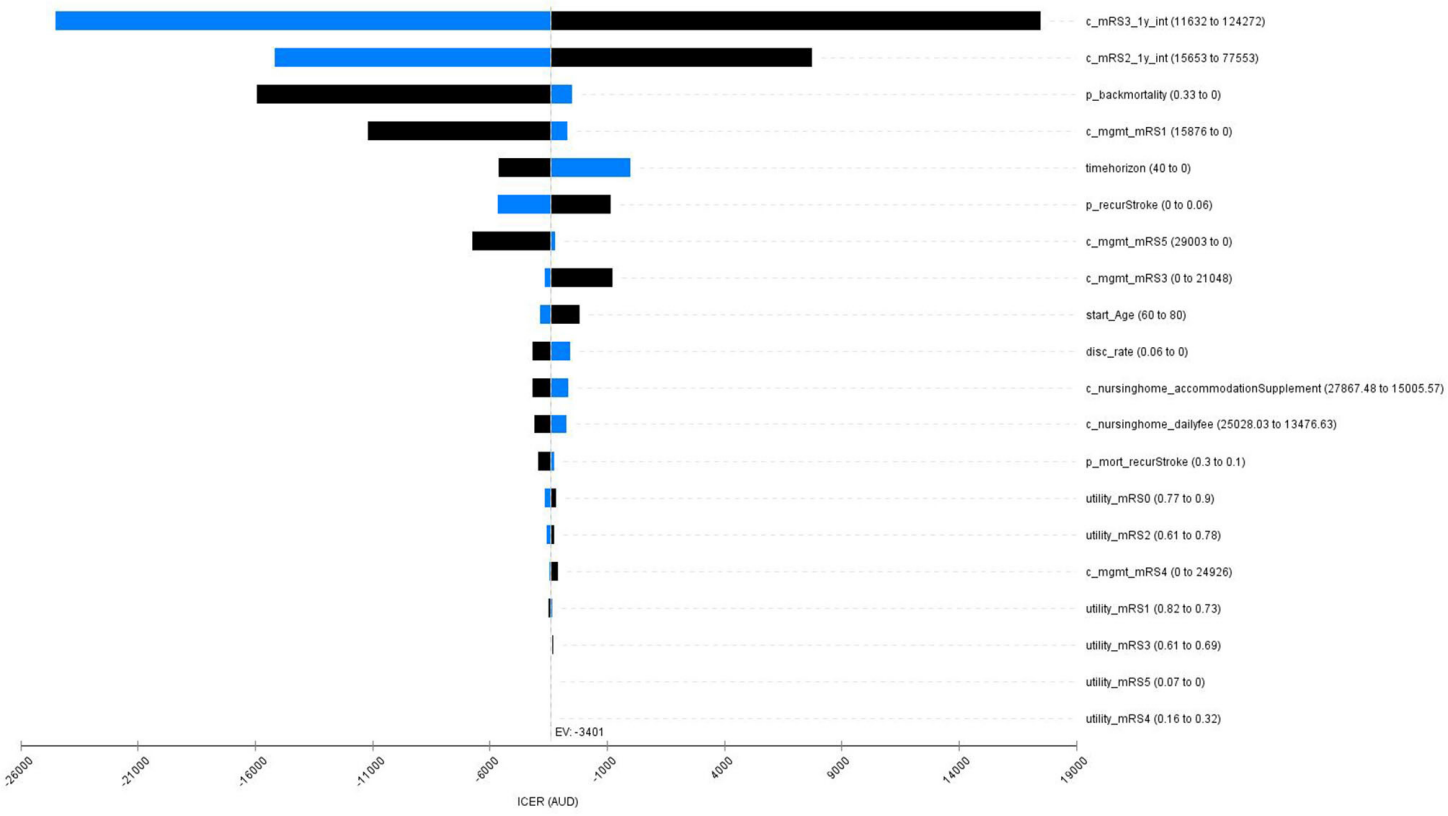
A Tornado diagram for the one-say sensitivity analyses. Red bar means the value increases from the base case; blue bar represents the value decreases from the base case. mRS, modified Rankin Scale; mgmt, management; mort, mortality; c_mRS3_1y_int, cost of management for the intervention group for the first year post stroke (mRS3); c_mRS2_1y_int, cost of mangement for the intervention group for the first year post stroke (mRS2); p_backmortality, probability of background mortality; c_mgmt_mRS1, cost of long-term management post stroke (mRS1); timehorizon, modeled time horizion; p_recurStroke, probability of having recurrent stroke; c_mgmt_mRS5, cost of long-term management post stroke (mRS5); c_mgmt_mRS3, cost of long-term management post stroke (mRS3); start_Age, onset age of the index stroke; disc_rate, discount rate for both costs and QALYs; c_nursinghome_accommodationSupplement, cost of nursing home care for the accommodation supplement; c_nursinghome_dailyfee, cost of nursing home care for the daily fee; utility_mRS0, utility weights post stroke (mRS0); utility_mRS2, utility weights post stroke (mRS2); c_mgmt_mRS4, cost of long-term management post stroke (mRS4); p_mort_recurStroke, probabiltiy of death following a recurrent stroke; utility_mRS1, utility weights post stroke (mRS1); utility_mRS3, utility weights post stroke (mRS3); utility_mRS5, utility weights post stroke (mRS5); utility_mRS4, utility weights post stroke (mRS4).

The one-way deterministic sensitivity analysis results showed that the base case results were robust to the variation in key model inputs (variation in all the inputs did not alter the conclusion of cost-effectiveness of the stroke telemedicine program). However, the changes in costs of the first-year stroke management (patients with the 3-months mRS scores of 1, 2, and 3), background mortality, time horizon, and probability of recurrent stroke contributed to the variation in ICER (Figure 2).

**Figure 3:**
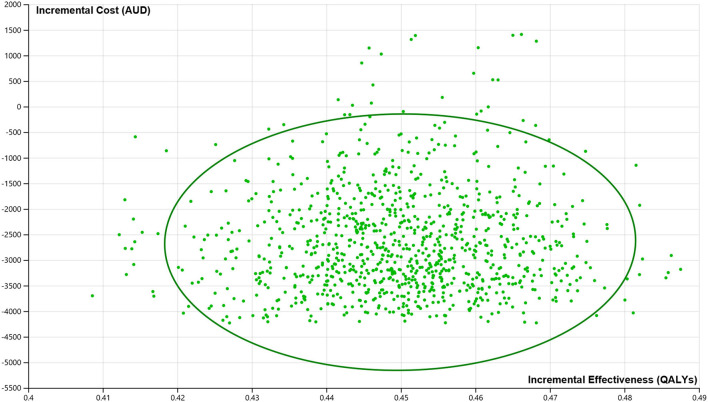
Incremental cost-effectiveness plane from the probabilistic sensitivity analysis. AUDs, Australian dollars; QALYs, quality-adjusted life years. 100% of all the iterations suggest that the stroke telemedicine program is cost saving and more effective over the lifetime of patients.

The PSAs showed that the probability of the stroke telemedicine program being the dominant option is high (100%) by incorporating the distribution of key variables (Figure 3).

The cutoff value for the population size to implement the cost-effective stroke telemedicine program was 67 (e.g., a region with a minimum 67 suspected strokes, equivalent to $26,312 per person, over 1 year could sustain the cost-effectiveness of the stroke telemedicine program) (Supplementary Figure 1).

### Impact of the National Implementation

Implementing the stroke telemedicine program Australia-wide was associated with cost savings and more health gains. A single year implementation could lead to a maximum of $19 million savings and 5,646 additional QALY gained over a lifetime compared to current practice where such a program does not exist. The savings could completely offset the implementation cost of $37 million from reduced need for nursing home care combined with reduced costs of acute hospitalization for the index stroke (Table 3).

**Table 3:** Results from the national implementation.

	National impact^*^		
	**Scenario 1 (20%)**	**Scenario 2 (50%)**	**Scenario 3 (100%)**
Total costs	–$1,985,002	–$3,114,400	–$4,996,729
Total QALYs	$2,243	$3,519	$5,646
Management cost	$4,062,273	$6,373,567	$10,225,722
Rehospitalisation cost	–$4,042,645	–$6,342,771	–$10,176,313
Nursing home cost	–$10,883,496	–$17,075,830	–$27,396,386
Non-medical cost	–$637,245	–$999,816	–$1,604,100
First 12-month cost	$9,516,111	$14,930,450	$23,954,349

* *Scenarios 1 to 3 assumed that 20% (n = 4997), 50% (n = 7840), and 100% (n = 12578) of inner regional population (plus 100% of outer regional population) would be eligible for the VST program of 38,055 stroke Australia-wide*.

*QALY, quality-adjusted life year*.

## Discussion

We provide evidence that the implementation of the national stroke telemedicine program for patients with ischemic stroke from regional Australia in the long term provides more health gains and cost savings from the societal perspective or is highly cost-effective (ICER of $1,736 per QALY gained) from the healthcare system perspective and is a worthwhile objective to achieve. The model-based cost-effectiveness analysis extending the short-term patient-level results to the long-term patient-level indicated that implementing the stroke telemedicine program in Victoria was associated with lower costs and greater health benefits from a societal perspective or higher costs and benefits from a healthcare system perspective. The cost savings exceeded the cost of implementing the stroke telemedicine program from any subsequent hospitalizations, nursing home care, and non–medical care. Moreover, the long-term model also suggested that the stroke telemedicine program could potentially avoid the need for nursing home care (101 cases avoided per 10,000 patients).

Our short-term individual patient-level data-based economic evaluation of the stroke telemedicine program reported that using telemedicine technology to diagnose and treat patients with acute stroke was most likely to be cost-effective at 12 months poststroke within Australia (7). However, during the acute phase of the index stroke, greater acute costs were observed for participants of the stroke telemedicine program, probably due to the increased frequency of transfer following specialist recommendations (averaging $1,866 per trip for people from regional/rural areas by Ambulance Victoria).

A recent systematic review of the economic evaluations (cost-effectiveness and cost-utility analyses) of telemedicine/telestroke identified eight economic evaluations worldwide (5). It was reported that there was heterogeneity in the design across the reviewed studies. It is worth mentioning that the cost-effectiveness of the stroke telemedicine program generally varied according to the time horizon of the analysis. For example, the high upfront costs of implementing such a program (i.e., infrastructure and equipment to deliver the services, etc., totaling over $1.8 million in the VST) cannot be offset by the subsequent cost saving from treatment/management in the short term. In the reviewed articles, cost savings arose primarily from decreased nursing home care required or avoided interhospital transfer.

The results from this long-term model-based cost-effectiveness analysis were comparable with prior long-term studies. Generally, all the studies found that the stroke telemedicine program improved quality of life outcomes [ranging from 0.02 (27) to 1.3 (28) QALYs gained; the incremental QALYs (0.449) from our modeling fell within this range]. However, the cost results were slightly inconsistent: incremental costs over short timeframes from the stroke telemedicine programs ranged between cost savings of US$ 4,241 (29) to additional costs of US$ 3,006 (30) per patient, while for long-term horizons, they varied from cost savings of US$ 19,888 (30) to an additional cost of US$ 3,184 (28) per patient.

In this study, the benefits from the stroke telemedicine program were mainly driven by the proportion of patients who arrived < 4.5 h from stroke onset and received intravenous thrombolysis (i.e., increased from 17 to 26%), which has a demonstrated effectiveness in reducing poststroke disability (6). The 12-month mRS outcomes post the index stroke were directly informed by patient-level data from the primary data collection. All the event rates, event-related costs, and utility weights were identical for both the stroke telemedicine and non–stroke telemedicine scenarios in the long-term model. The disability avoided by timely intravenous thrombolysis was then extrapolated over the remaining life course. The potential benefit to the stroke telemedicine program of endovascular thrombectomy (EVT), which was introduced in Australia in 2015, was not fully captured in the current data collection (due to time lag). Large vessel occlusion (LVO) stroke, although only accounting for 10–15% of all the strokes, represents a disproportionate share of the disease burden. Without EVT, LVO stroke is associated with more than 50% mortality or major disability at 3 months (31). The stroke telemedicine program provides rapid access to stroke specialists. Thus, eligible patients can be accurately identified and transferred in a timely manner to a metropolitan hospital with EVT capability (only four metropolitan hospitals have the facility for EVT procedure 24/7 in Victoria); the milestone trials have demonstrated that EVT led to the significantly higher rate of functional independence and decreased mortality at the 3-month post the index stroke (32–38). It is highly probable that the potential cost saving and health benefits from the stroke telemedicine program would be further expanded if the combined benefits from both the intravenous thrombolysis and endovascular thrombectomy could have been measured.

In, 2017–2018, the Australian government spent over $18 billion on aged care with 67% of total expenditure on residential care ($12 billion) (39). This study indicates that when modeled for the national population, the stroke telemedicine program was associated with substantial savings in terms of nursing home care. It is also worth noting that the productivity cost was not included in the current long-term model due to the mean age of the simulated population. However, a recent report on the burden of heart valve disease in Australia suggests that the elderly population contributes substantially to the economy through unpaid productivity by providing childcare for grandchildren, caring for family members, and volunteering (40). The cost saving of the stroke telemedicine program from a societal perspective could be further increased if such unpaid productivity costs were considered.

This is the first study in Australia that translated the short-term benefits of a stroke telemedicine program into long-term cost savings and health outcomes. The patient-level data directly informed the costs and outcome for the first year following program implementation. However, this study is not without limitations. First, the long-term model only simulated the events related to recurrent stroke, whereas, in reality, other cardiovascular (heart attack) or non–cardiovascular (such as cancer) events may occur. This does not favor the intervention under evaluation given the higher probability of other cardiovascular events with advanced disability poststroke (41). Second, it was assumed that the functional status at 12-months poststroke could not be improved (i.e., patients with the mRS 3 cannot enhance to mRS 2) over the remaining life course, which is not necessarily the case. Third, while the cost-effectiveness results from this study cannot be directly transferred to another jurisdiction for funding decision-making, they can be adapted (with local costs and utility weights) for timely use. Lastly, the results from the long-term model are likely to be conservative, given not all the benefits related to the stroke telemedicine service were able to be captured. In particular, patients with suspected stroke get earlier diagnosis and treatment, if being hemorrhagic or stroke mimics. In addition, intangible benefits from better coordination of stroke care, improved capacity building through enhanced training and experience, reduced caregiver burden from lowered transport costs, and improved quality of life are not measured and valued in the current estimation. Also, since the stroke incidence is projected to increase (42), the benefit from the national implementation may have been underestimated. The cost of potentially more treatment (i.e., transferring patients timely for thrombectomy) due to the application of the stroke telemedicine program was not incorporated in the current estimation due to the study timeframe (2010–2016). Moreover, the change in the cost of thrombolysis and telemedicine staff salaries is not factored into account.

## Conclusion

The findings from this long-term model provide evidence to support ongoing funding for stroke telemedicine services in Australia. Our estimates are conservative since other benefits of the service outside the use of intravenous thrombolysis were not included, e.g., optimal management of patients with intracerebral hemorrhage.

## Data Availability Statement

The data that support this study cannot be publicly shared due to ethical or privacy reasons and may be shared upon reasonable request to the corresponding author if applicable.

## Ethics Statement

The studies involving human participants were reviewed and approved by Monash University Human Research Ethics Committee (2014-2482-2291). The patients/participants provided their written informed consent to participate in this study.

## Author Contributions

LG, DC, and MM designed the study. LG, ET, and JK undertook the analysis. LG, ET, JK, DC, and MM interpreted the results. LG drafted the manuscript. LG, ET, JK, CB, HD, KB, DC, and MM provided critical input for the manuscript. All authors contributed to the article and approved the submitted version.

## Funding

A National Health and Medical Research Council project grant (1079179) was received to enable a comprehensive economic evaluation of the VST program. We received financial support for the VST project from the Windermere Foundation, the Victorian Departments of Business and Innovation and of Health, and the Australian Government. The Florey Institute of Neuroscience and Mental Health acknowledges the support of the Victorian Government, particularly with an Operational Infrastructure Support Grant. DC was supported by National Health and Medical Research Council research fellowships paid to her institution during the study (1063761 co-funded Heart Foundation; 1154273). CB and DC have received unrestricted educational grants from Boehringer Ingelheim for the VST project.

## Conflict of Interest

The authors declare that the research was conducted in the absence of any commercial or financial relationships that could be construed as a potential conflict of interest.

## Publisher's Note

All claims expressed in this article are solely those of the authors and do not necessarily represent those of their affiliated organizations, or those of the publisher, the editors and the reviewers. Any product that may be evaluated in this article, or claim that may be made by its manufacturer, is not guaranteed or endorsed by the publisher.
