# Supplementary material for: Telemedicine for Stroke: Quantifying the Long-Term National Costs and Health Benefits
Source: Front Neurol. 2022 Jun 20;12:804355. doi: 10.3389/fneur.2021.804355 (PMC9265143; doi:10.3389/fneur.2021.804355)
Supplement: Supplementary file 1 [file Data_Sheet_1.pdf]

Supplementary material

Supplementary Table 1. Costs inputs from the Victorian Stroke Telemedicine program study

	Mean	Lower CI	Upper CI	Mean	Lower CI	Upper CI
Cost of acute hospitalisation						
	No Telestroke			Telestroke		
mRS 0	$8,560	$6,444	$10,675	$12,643	$10,702	$13,675
mRS 1	$11,361	$8,648	$14,074	$15,261	$12,856	$17,085
mRS 2	$21,575	$16,807	$26,343	$25,278	$21,569	$28,016
mRS 3	$32,293	$26,881	$37,705	$35,851	$30,433	$40,430
mRS 4	$42,503	$32,708	$52,298	$51,055	$42,079	$58,864
mRS 5	$47,108	$31,043	$63,173	$49,390	$33,541	$62,129
mRS 6	$12,260	$10,992	$13,529	$15,589	$13,265	$16,985
Cost of first 12-month						
	No Telestroke			Telestroke		
mRS 0	$39,851			$36,601		
mRS 1	$52,619	$8,499	$96,740	$32,409	$10,622	$52,743
mRS 2	$60,276	$10,248	$110,304	$47,456	$15,653	$77,553
mRS 3	$64,101	$13,635	$114,566	$68,228	$11,632	$124,272
mRS 4	$89,374	$14,954	$163,793	$92,415	$38,839	$150,527
mRS 5	$76,799	$9,141	$144,457	$82,837	$6,001	$159,611
mRS 6	$15,970			$11,769		

Abbreviations: CI: confidence interval; mRS: modified Rankin scale; telestroke: stroke telemedicine program.

Supplementary Table 2. Parameters of distributions examined in the probabilistic sensitivity analyses

Variable	Distribution in PSA	Reference
Cost of stroke telemedicine program	Gamma distribution (alpha 3.039; lambda 0.0017)	Australian Telestroke Network study
HR for background mortality (mRS 4)	Gamma distribution (alpha 100; lambda 58.48)	Samsa et al. 1999[45]
Utility weight for mRS 1	Beta distribution (alpha 245.05; beta 70.29)	Kim et al 2021[7]
Utility weight for mRS 2	Beta distribution (alpha 95.21; beta 41.92)	
Utility weight for mRS 3	Beta distribution (alpha 77.28; beta 99.52)	

Abbreviations: HR: hazard ratio; mRS:modified Rankin scale; PSA: probabilistic sensitivity analysis
